# Maternal migraine, vascular comorbidities, and prematurity: exploring overlooked associations in pregnancy

**DOI:** 10.3389/fgwh.2026.1777662

**Published:** 2026-07-02

**Authors:** Milan Lackovic, Dejan Nikolic, Sladjana Mihajlovic, Bojana Ivic, Aygul Izhanova Kuandykovna, Araily Manasbayeva Elubaevna, Jovana Kuzmanovic Pficer

**Affiliations:** 1 Harris Birthright Research Centre for Fetal Medicine, King’s College Hospital, London, United Kingdom; 2 Department of Obstetrics and Gynecology, University Hospital “Dragisa Misovic”, Belgrade, Serbia; 3 Department of Physical Medicine and Rehabilitation, University Children’s Hospital, Belgrade, Serbia; 4 Faculty of Medicine, University of Belgrade, Belgrade, Serbia; 5 Clinic for Gynecology and Obstetrics “Narodni Front”, Belgrade, Serbia; 6 Department of Internal Medicine, Asfendiyarov Kazakh National Medical University, Almaty, Kazakhstan; 7 Department of Children’s Diseases Named After Professor N.A. Barlybaeva, Asfendiyarov Kazakh National Medical University, Almaty, Kazakhstan; 8 Department of Medical Statistics and Informatics, School of Dental Medicine, University of Belgrade, Belgrade, Serbia

**Keywords:** migraine, obesity, pregnancy, pregnancy complications, prematurity

## Abstract

**Background:**

Prematurity remains a leading cause of neonatal morbidity and mortality, with lasting implications for neurodevelopment and lifelong health. This study aimed to investigate maternal and fetal factors associated with preterm birth within a cohort of pregnant women affected by migraine, with particular focus on vascular and metabolic comorbidities potentially contributing to adverse pregnancy outcomes.

**Methods:**

We performed prospective cohort study. Pregnant women presenting in the first trimester (≤11 weeks gestation) with a history of migraine prior to conception and at least one migraine attack during the first trimester were enrolled. Participants underwent neurological evaluation to exclude secondary headache disorders and completed structured questionnaires on migraine frequency and intensity pregestation and during pregnancy. Participants were stratified by gestational age at delivery into preterm (<37 weeks) and term (≥37 weeks) groups. Maternal, fetal, and pregnancy-related variables assessed included maternal age, parity, maternal obesity, gestational weight gain, fetal growth restriction, delivery mode, pregnancy-induced hypertension, and gestational diabetes mellitus.

**Results:**

A total of 400 women were included, of whom 65 delivered preterm. Statistically significant associations with preterm delivery were observed for pregnancy-induced hypertension (*p* < 0.001), gestational diabetes mellitus (*p* < 0.001), maternal obesity (*p* = 0.021), excessive gestational weight gain (*p* < 0.001), fetal growth restriction (*p* < 0.001), parity (*p* < 0.001), delivery mode (*p* < 0.001), and migraine frequency (*p* = 0.015) and intensity (*p* = 0.020) worsening during pregnancy. Excessive gestational weight gain emerged as a key modifiable risk factor. Women who delivered preterm reported more frequent and intense migraine attacks compared with term deliveries.

**Conclusions:**

Our findings support the hypothesis that migraine is a systemic vascular condition with significant implications for pregnancy outcomes. The coexistence of migraine with pregnancy-induced hypertension, gestational diabetes mellitus and maternal obesity suggests that shared pathophysiological mechanisms may increase the risk of prematurity. While pre-pregnancy obesity alone was not an independent predictor, gestational weight gain represents a critical modifiable factor. These results underscore the need for individualized, multidisciplinary prenatal care for women with migraine, emphasizing vascular risk assessment, blood pressure monitoring, weight management, and optimized migraine control. Future studies are warranted to clarify causal mechanisms and develop safe therapeutic strategies to protect both maternal and fetal health.

## Background

Prematurity remains one of the leading causes of early neonatal morbidity and mortality and exerts profound, long-lasting effects related to chronic disease, lifelong risk of disability and neuromotor development of affected offspring (1). Although substantial improvements in neonatal survival among preterm infants have been achieved over recent decades, the enduring consequences associated with the early, premature disruption of the maternal–fetal interface continue to pose a major, and often insurmountable, challenge throughout the lifespan (2). Despite intensive global efforts, the overall rate of preterm birth has remained relatively stable, affecting approximately 10% of all deliveries worldwide (3). Considerable resources have been invested in prevention and management strategies, however, evidence suggesting meaningful progress toward mitigating this critical and life-threatening condition remains limited globally, especially in regions with constrained healthcare infrastructure (2). Moreover, emerging data from industrialized, high-income countries indicate that, although modest reductions in spontaneous preterm birth have been observed, the rates of iatrogenic preterm birth continue to rise (4, 5).

Maternal health conditions that compromise women's well-being and, in more severe cases, threaten maternal life, constitute the leading contributors necessitating iatrogenic prematurity. Chronic maternal diseases persist as major drivers of medically indicated early delivery, underscoring the vulnerability of the unique, intricate biological relationship between mother and fetus (6). Throughout the typical 40 weeks of gestation, the fetus acquires essential physiological competencies that prepare it for extrauterine, independent life. The intrauterine environment provides a secure and finely regulated setting that supports the most complex and subtle phase of human development, transforming a single fertilized cell into a fully functional organism capable of sustaining independent life. Advances in neonatal intensive care have dramatically expanded the limits of ex-utero viability, with survival now reported at gestational ages as low as 22–23 weeks. While this represents a remarkable milestone in modern medicine, substantial variations regarding survival rates between different centers have been reported, and furthermore a wide range of new neonatal challenges and potential long-term complications requiring careful consideration has been introduced (7, 8).

Migraine is a highly prevalent neurological disorder that disproportionately affects women, particularly those of reproductive age (9). Despite its substantial disease burden and well-established associations with chronic medical conditions, including cardiovascular and metabolic disorders, the severity of migraine and its profound impact on overall health often remains underrecognized (10, 11). Emerging evidence suggests an association between migraine and an increased risk of adverse perinatal outcomes (12). Although the underlying mechanisms linking migraine to pregnancy complications are not yet fully elucidated, growing data indicate that altered vascular reactivity, endothelial dysfunction, hypercoagulability, and systemic inflammation play central roles. As pregnancy progresses, the physiological demands placed on maternal cardiovascular and metabolic systems intensify. In this context, compromised maternal adaptive mechanisms associated with migraine may be insufficient to meet the escalating demands of gestation, thereby predisposing affected women and her unborn child to adverse outcomes (13, 14). Accordingly, migraine may represent an important and underappreciated risk factor for pregnancy complications, potentially interfering with the complex physiological adaptations required during gestation, often regarded as one of the most significant physiological stress tests in a woman's life. Importantly, migraine frequently coexists with vascular and metabolic conditions such as obesity, hypertensive disorders, and impaired glucose metabolism, all of which are independently associated with adverse obstetric outcomes and potential long-term consequences for offspring (15–17). Therefore, understanding pregnancy outcomes among women with migraine requires consideration of these interconnected maternal factors rather than interpretation of migraine as an isolated determinant of prematurity.

Given the high prevalence of migraine among women of reproductive age and its reported associations with prematurity and other pregnancy-related complications, heightened clinical awareness, careful antenatal surveillance, and further research are warranted to elucidate underlying causal mechanisms and identify effective preventive strategies. Determining whether migraine increases vulnerability to adverse pregnancy outcomes is therefore of substantial clinical relevance. Accordingly, the aim of this study was to investigate the potential association between migraine and prematurity, a major concern in perinatology and neonatology.

## Methods

### Study population and recruitment

Participants were recruited during the first trimester of pregnancy, no later than the 11th gestational week. Eligible participants were pregnant women who reported at least one migraine attack within the three months preceding conception and at least one migraine attack during the first trimester of pregnancy. Recruitment was conducted over a two-year period, and it started in the beginning of 2024. Study took place at the Department of Obstetrics and Gynecology, University Hospital “Dragisa Misovic” in Belgrade, Serbia.

To exclude secondary causes of headache, all participants underwent a comprehensive neurological examination. Migraine diagnosis was established according to the International Classification of Headache Disorders, 3rd edition (ICHD-3) (18), developed by the International Headache Society (IHS) diagnostic criteria (19).

### Data collection

All participants completed a structured questionnaire designed to collect detailed information regarding the frequency and intensity of migraine attacks before and during pregnancy. The questionnaire was administered on two occasions: during the first prenatal visit and again three months postpartum. Data used for analysis were extracted from the completed questionnaires and from the patients hospital medical records.

### Ethical considerations

Prior to enrollment, all participants provided written informed consent. The study protocol was conducted in accordance with ethical principles for medical research involving human subjects. Study obtained Institutional Review Board (IRB) approval (No. 11297/3, Date: 27th of May 2024).

### Inclusion and exclusion criteria

Women younger than 18 years of age were excluded from the study. Additional exclusion criteria included multifetal pregnancies and the presence of chronic maternal medical conditions other than migraine.

### Study group allocation

To address the study objectives, participants were stratified based on gestational age at delivery. Preterm delivery was defined as birth occurring before 37 completed weeks of gestation. Accordingly, participants were initially divided into two groups: those who delivered preterm (<37 weeks) and those who delivered at term (≥37 weeks). The study was designed to explore factors associated with preterm birth among pregnant women affected by migraine rather than to establish migraine as an independent risk factor compared with a non-migraine population.

### Study variables

Fourteen study variables were assessed, including maternal age and advanced maternal age (>35 years); age at migraine onset; migraine frequency and intensity before and during pregnancy; parity; pre-pregnancy obesity [defined as body mass index (BMI) > 30 kg/m^2^]; gestational weight gain (GWG) [categorized according to Institute of Medicine (IOM) guidelines (20): insufficient or excessive if below or above cut-off values]; delivery mode (vaginal vs. cesarean section); fetal growth restriction [defined as estimated fetal weight <10th percentile, fetal abdominal circumference <10th percentile, or evidence of placental insufficiency via abnormal Doppler studies (21)]; birth weight >90th percentile; percentile distribution of estimated fetal weight by gestational age; pregnancy-induced hypertension (PIH), including gestational hypertension, preeclampsia, eclampsia, or HELLP syndrome; gestational diabetes mellitus (GDM), diagnosed according to American Diabetes Association (ADA) guidelines (22). Migraine symptom changes during pregnancy were classified into three groups based on self-reported frequency and intensity: (1) improved or resolved, (2) unchanged, and (3) worsened.

### Statistical analysis

Descriptive statistics and comparisons between groups were performed in SPSS version 22 (IBM SPSS Statistics, version 22.0; IBM Corp., Armonk, NY, USA) using the chi-square tests. Fisher's exact test was applied when expected cell counts were small, while Pearson's chi-square test was used for larger samples. Data are presented as absolute values ​​and percentages. Advanced analyses of multivariate logistic models were performed in the R software package version 4.5.1; the glmnet, caret, and pROC packages were used for LASSO penalized regression, while the logistf package was used for Firth penalized logistic regression. A two-stage analysis was used to identify independent factors associated with preterm birth. Given the relatively small number of events (*n* = 65) compared to the number of potential predictors (*n* = 10), LASSO-penalized logistic regression was first applied to select predictors and reduce overfitting. Coefficients that were nonzero were considered relevant for further analysis. The model's performance was evaluated using prediction accuracy, AUC (area under the ROC curve), and regularization (*λ*). After factor selection, the final inferential model, Firth's penalized logistic regression, was used to determine predictors of preterm birth. The results are presented as odds ratios (OR) with 95% confidence intervals, and the significance of predictors was assessed by the likelihood ratio test.

## Results

In Table 1, distribution of tested variables are presented. The mean age of tested participants was 31.10 ± 6.20 years. Most frequent number of deliveries was one (*n* = 211, 52.8%). Study subjects were more frequently younger than 35 (*n* = 285, 71.3%), had body mass index lower than 30 (*n* = 267, 66.9%) and normal gestational weigh gain (*n* = 243, 60.8%). Vaginal mode of delivery was more frequent (*n* = 282, 70.5%), most of the study subjects did not have pregnancy-induced hypertension (*n* = 330, 82.5%) or gestational diabetes mellitus (*n* = 310, 77.5%). Fetal growth was normal in majority of cases (321, 80.3%), and in the majority of cases fetal growth restriction or birth weight >90th percentile was not detected (*n* = 346, 86.5%) and (*n* = 376, 94.0%) respectively. Migraine pain intensity during pregnancy vs. before pregnancy most frequently increased (*n* = 148, 37.0%), while migraine frequency during pregnancy vs. before pregnancy most frequently remained unchanged (*n* = 178, 44.5%).

**Table 1:** Distribution of evaluated variables.

Variables		Value
Age, years (MV ± SD)		31.10 ± 6.20
Number of deliveries, *n* (%)	1	211 (52.8%)
	2	147 (36.8%)
	3	41 (10.3%)
	4	1 (0.3%)
Number of deliveries (MV ± SD)	1.58 ± 0.68	
Gestational age >35 GW, *n* (%)	No	285 (71.3%)
	Yes	115 (28.7%)
BMI, *n* (%)	<30	267 (66.9%)
	≥30	132 (33.1%)
GWG, *n* (%)	Normal	243 (60.8%)
	Above	125 (31.3%)
	Below	32 (8.0%)
Age at migraine onset (MV ± SD)	15.53 ± 3.90	
PIH, *n* (%)	No	330 (82.5%)
	Yes	70 (17.5%)
GDM, *n* (%)	No	310 (77.5%)
	Yes	90 (22.5%)
Migraine pain intensity during pregnancy vs. before pregnancy, *n* (%)	Decreased	113 (28.2%)
	Unchanged	139 (34.8%)
	Increased	148 (37.0%)
Migraine frequency during pregnancy vs. before pregnancy, *n* (%)	Decreased	114 (28.5%)
	Unchanged	178 (44.5%)
	Increased	108 (27.0%)
Preterm delivery, *n* (%)	Yes	65 (16.3%)
	No	335 (83.8%)
Mode of delivery, *n* (%)	Vaginal	282 (70.5%)
	Induction	115 (28.7%)
Fetal growth restriction, *n* (%)	No	346 (86.5%)
	Yes	54 (13.5%)
Fetal growth, *n* (%)	Below 10%	30 (7.5%)
	Below 5%	18 (4.5%)
	Below 3%	8 (2.0%)
	Above 90%	23 (5.8%)
	Normal growth	321 (80.3%)
LGA, *n* (%)	No	376 (94.0%)
	Yes	24 (6.0%)

GW, gestational week; BMI, body mass index; GDM, gestation diabetes mellitus; GWG, gestational weight gain; PIH, pregnancy induced hypertension; LGA, large for gestational age.

The distribution of the studied variables with respect to preterm delivery is presented in Table 2. Pre-pregnancy obesity (*p* = 0.021) and excessive gestational weight gain (*p* < 0.001) were significantly more frequent among women who delivered preterm. In this group, pregnancy-induced hypertension (*p* < 0.001), gestational diabetes mellitus (*p* < 0.001), and fetal growth restriction (*p* < 0.001) were also significantly more common. Furthermore, women with preterm delivery showed a significantly higher increase in migraine pain intensity during pregnancy compared with the pre-pregnancy period (*p* = 0.020), as well as a higher migraine frequency during pregnancy vs. pre-pregnancy (*p* = 0.015). Caesarean section was more frequent in the preterm delivery group (*p* < 0.001), and these women had lower parity (*p* < 0.001).

**Table 2:** Distribution of tested variables regarding preterm delivery.

Variables		Preterm delivery		*p* value
		No	Yes	
Age, years (MV ± SD)		30.95 ± 6.23	31.86 ± 6.00	0.208^a^
Number of deliveries (MV ± SD)		1.64 ± 0.69	1.29 ± 0.55	<0.001^a^
Gestational age >35 GW, *n* (%)	No	240 (71.6%)	45 (69.2%)	0.765^b^
	Yes	95 (28.4%)	20 (30.8%)	
BMI, *n* (%)	<30	232 (69.5%)	35 (53.8%)	0.021^b^
	≥30	102 (30.5%)	30 (46.2%)	
GWG, *n* (%)	Normal	228 (68.1%)	15 (23.1%)	<0.001^c^
	Above	93 (27.8%)	32 (49.2%)	
	Below	14 (4.2%)	18 (27.7%)	
Age at migraine onset (MV ± SD)		15.53 ± 3.75	15.49 ± 4.62	0.280^a^
PIH, *n* (%)	No	316 (94.3%)	14 (21.5%)	<0.001^b^
	Yes	19 (5.7%)	51 (78.5%)	
GDM, *n* (%)	No	277 (82.7%)	33 (50.8%)	<0.001^b^
	Yes	58 (17.3%)	32 (49.2%)	
Migraine pain intensity during pregnancy vs. before pregnancy, *n* (%)	Decreased	99 (29.6%)	14 (21.5%)	0.020^c^
	Unchanged	122 (36.4%)	17 (26.2%)	
	Increased	114 (34.0%)	34 (52.3%)	
Migraine frequency during pregnancy vs. before pregnancy, *n* (%)	Decreased	100 (29.9%)	14 (21.5%)	0.015^c^
	Unchanged	154 (46.0%)	24 (36.9%)	
	Increased	81 (24.2%)	27 (41.5%)	
Mode of delivery, *n* (%)	Vaginal	253 (75.5%)	30 (46.2%)	<0.001^c^
	Induction	82 (24.5%)	34 (52.3%)	
Fetal growth restriction, *n* (%)	No	322 (96.1%)	24 (36.9%)	<0.001^b^
	Yes	13 (3.9%)	41 (63.1%)	
Fetal growth, *n* (%)	Below 10%	13 (3.9%)	17 (26.2%)	<0.001^c^
	Below 5%	1 (0.3%)	17 (26.2%)	
	Below 3%	1 (0.3%)	7 (10.8%)	
	Above 90%	18 (5.4%)	5 (7.7%)	
	Normal growth	302 (90.1%)	19 (29.2%)	
LGA, *n* (%)	No	316 (94.3%)	60 (92.3%)	0.566^b^
	Yes	19 (5.7%)	5 (7.7%)	

GW, gestational week; BMI, Body Mass Index; GDM, Gestation Diabetes Mellitus; GWG, Gestational Weight Gain; PIH, pregnancy induced hypertension; LGA, large for gegstational age.

a Mann–Whitney U test.

b Fishers Exact Test.

c Chi Square Test.

Multivariate Lasso regression analysis included 10 potential independent factors: parity, pre-pregnancy obesity, gestational weigh gain, pregnancy-induced hypertension, gestational diabetes mellitus, migraine pain intensity and frequency during pregnancy vs. pre-pregnancy, deliver mode, fetal growth restriction and percentile distribution of estimated fetal weight by gestational age.

**Figure 1:**
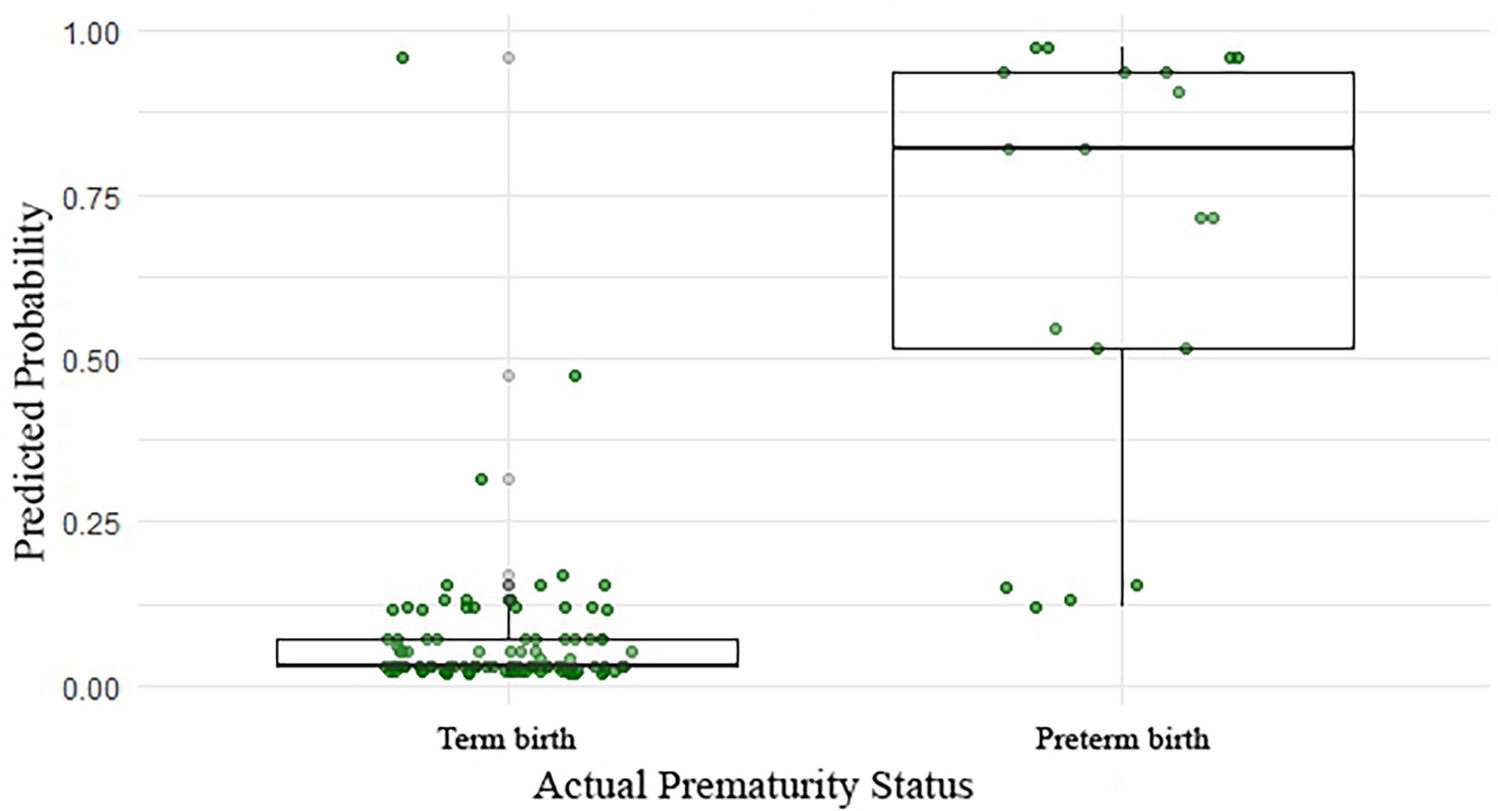
LASSO Model: Predicted probability by actual preterm birth Class.

The results show that after Lasso selection, the following variables remained in the model: parity, gestational weigh gain, pregnancy-induced hypertension, gestational diabetes mellitus, fetal growth restriction and percentile distribution of estimated fetal weight by gestational age. The model demonstrated exceptional ability to distinguish preterm from term births in the test set. The prediction accuracy was 95.8%, while the AUC value was 0.976, and the optimal regularization value (*λ*) determined through cross-validation was 0.0139. Also, Figure 1, showed that boxplots demonstrate lower predicted probabilities for term birth and higher predicted probabilities for preterm birth, indicating good discriminative performance of the LASSO model.

The following variables were independently associated with preterm birth in the multivariate Firth logistic regression model. Pregnancy-induced hypertension was strongly associated with an increased risk of preterm delivery (OR = 26.58; 95%CI: 9.51–82.71; *p* < 0.001). Also, gestational diabetes mellitus significantly increased the risk (OR = 4.56; 95%CI: 1.71–13.19; *p* = 0.002). Excessive gestational weight gain was associated with a higher risk of preterm delivery (OR = 3.89; 95%CI: 1.44–11.21; *p* = 0.007). The number of deliveries showed borderline statistical significance (OR = 0.51; 95% CI: 0.24–1.01; *p* = 0.054), suggesting a potential protective effect. Fetal growth restriction did not reach statistical significance but showed a trend towards an increased risk (OR = 15.39; 95% CI: 0.71–258.32; *p* = 0.084). The variable percentile distribution of estimated fetal weight by gestational age was not significantly related to the outcome (*p* = 0.993). The model as a whole was statistically significant (likelihood ratio test: *χ*^2^ = 205.63; df = 10; *p* < 0.001) (Table 3).

**Table 3:** Firth'S penalized logistic regression analysis of predictors of preterm birth.

Variables	Odds Ratio	95% CI	*p* values
Number of deliveries	0.51	0.24–1.01	0.054
GWG – above	3.89	1.44–11.21	0.007
GWG – below	0.75	0.15–3.44	0.717
PIH	26.58	9.51–82.71	<0.001
GDM	4.56	1.71–13.19	0.002
Fetal growth	1.70	0.12–35.67	0.993
Fetal growth restriction	15.39	0.71–258.32	0.084

GWG, gestational weight gain; GDM, gestational diabetes mellitus; CI, confidence interval; Referent category: GWG, normal; PIH, pregnancy induced hypertension.

## Discussion

The study comprised a total of 400 pregnant women diagnosed with migraine prior to conception, all of whom experienced at least one migraine attack during the first trimester of pregnancy. Participants were stratified into two groups based on gestational age at delivery: a preterm delivery group (<37 completed weeks of gestation) and a term delivery group (≥37 weeks of gestation). The preterm group included 65 participants, whereas 335 participants delivered at term.

Among the variables assessed for study purposes, statistically significant associations were identified between preterm delivery and several maternal and fetal factors within this migraine cohort, including pregnancy-induced hypertension (PIH), gestational diabetes mellitus (GDM), maternal pre-pregnancy obesity, excessive/insufficient gestational weight gain (GWG), fetal growth restriction (FGR), percentile distribution of estimated fetal weight by gestational age, parity, delivery mode and migraine symptom changes pregestational vs. during pregnancy.

Furthermore, multivariate Lasso regression analysis statistical demonstrated that pregnancy-induced hypertension, gestational diabetes mellitus and excessive gestational weight gain were independent predictors of prematurity among pregnant women affected by migraine.

The complex interplay between migraine and cardiovascular system function has been a subject of longstanding scientific interest. Endothelial dysfunction, chronic low-grade inflammation, vascular hyperreactivity, and a prothrombotic state are well-recognized features of migraine pathophysiology and may collectively impair maternal cardiovascular adaptive mechanisms during pregnancy (13). In this context, the relatively high prevalence of pregnancy-induced hypertension observed among our study population is not unexpected. Notably, the strong association between PIH and preterm delivery in our cohort further underscores the concept of heightened maternal vascular vulnerability in pregnant women affected by migraine (23).

The placenta represents a uniquely dynamic organ whose development relies on tightly regulated angiogenic processes to establish an extensive vascular network capable of sustaining fetal growth while facilitating metabolic exchange and waste elimination (24). Disruption of placental vascular function is a central mechanism underlying major obstetric complications, including hypertensive disorders of pregnancy, placental abruption, fetal growth restriction, and preterm birth (25). Given the vascular nature of migraine, it is plausible that chronic migraine-associated endothelial and vascular alterations (26) may potentially contribute to impaired placental perfusion. In this regard, the placenta may represent a potential target of systemic vascular dysregulation associated with migraine, rendering it particularly susceptible to maladaptive vasoreactivity during pregnancy.

In addition, both microvascular and macrovascular complications are well-established hallmarks of diabetes mellitus, contributing to long-term morbidity through effects on renal, retinal, and cardiovascular systems (27). In our previous research, we emphasized the potential synergistic effects of migraine with hypertensive disorders of pregnancy, gestational diabetes mellitus and obesity, proposing the concept of a “vascular triad” in which these conditions interact and amplify underlying vascular dysfunction (28). Migraine is frequently a chronic condition persisting throughout a woman's reproductive years, leaving a lasting imprint on physical and emotional health (29). GDM confers a lifelong risk since the incidence of progression to type 2 diabetes mellitus remains linear (30), while pregnancy-induced hypertension, particularly preeclampsia, is increasingly recognized as risk factors of future chronic hypertension (31). Collectively, these conditions appear to converge on shared pathophysiological pathways, with a prominent role of oxidative stress in obstetrics-related conditions, that ultimately increase long-term cardiovascular disease risk, the leading cause of morbidity and mortality worldwide (32).

The central role of placental angiogenesis further supports this integrated vascular hypothesis. The rapid and complex formation of placental vascular anastomoses during pregnancy inherently predisposes to developmental imperfections, which may manifest clinically as pregnancy complications (33, 34). The profound morbidity observed in monochorionic twin pregnancies due to placental anastomotic imbalance illustrates the critical importance of precise vascular regulation (35). We speculate that the coexistence of migraine, hypertensive disorders, and GDM may contribute to increased susceptibility to clinically relevant placental dysfunction, thereby increasing the risk of adverse perinatal outcomes, including prematurity.

Conversely, sex hormones are known to exert protective effects on the female cardiovascular system (36). Pregnancy and postpartum period represents a state of profound hormonal change, characterized by sustained elevations in estrogen and progesterone levels (37). In this context, the borderline protective association observed between increasing parity and prematurity in our study may reflect beneficial consequences of prolonged exposure to the vasoprotective effects of these hormones. Enhanced endothelial function and improved systemic vascular performance associated with repeated pregnancies could partially mitigate the deleterious vascular effects of migraine, thereby conferring a degree of protection against adverse pregnancy outcomes. On the other hand, nulliparous women are known to be at higher risk of spontaneous preterm delivery (38), and accordingly our conclusions cannot be supported only based on the possible protective role of prolonged sex specific hormone exposure during women's life.

The relationship between obesity and migraine remains controversial, with conflicting evidence regarding its potential protective or deleterious effects. Although maternal obesity is generally associated with adverse maternal and perinatal outcomes (39), some studies have reported a paradoxical protective effect against preterm birth (PTB) (40). In a secondary analysis of data from 20 centers in Brazil, Pigatti Silva et al. demonstrated that pre-pregnancy obesity was more strongly associated with iatrogenic PTB, whereas the incidence of spontaneous PTB was lower among obese women (41). In our cohort, obesity was more prevalent among women who delivered preterm; however, it did not emerge as an independent predictor of prematurity. In contrast, gestational weight gain (GWG) showed a strong positive association with PTB and represented one of the key findings of this study. We have previously highlighted the detrimental effects of excessive GWG on maternal, fetal, and neonatal outcomes (42), and the present results suggest that excessive GWG may also modulate and play an important role in the complex relationship between migraine and prematurity.

Women living with migraine in our cohort appeared to be particularly susceptible to obesity. Approximately half of the women who delivered preterm were obese, while more than two-thirds of those who delivered at term were obese. These findings revisit the concept of a paradoxical protective effect of pre-pregnancy obesity (40), while simultaneously underscoring the substantial impact of excessive GWG on pregnancy outcomes. Notably, fewer than one-quarter of women who delivered preterm achieved the IOM recommended GWG goals, compared with nearly two-thirds of women who delivered at term. Nearly 50% of women in the preterm group experienced excessive GWG, and an additional quarter had insufficient GWG, indicating that approximately three-quarters of women with PTB fell outside the recommended GWG range. By comparison, 69.1% of women with term deliveries met the IoM recommendations for GWG.

Furthermore, meta-analysis has confirmed that insufficient GWG is more frequently associated with spontaneous PTB (43), whereas Durts et al. have linked excessive GWG to an increased overall risk of PTB, particularly medically indicated PTB, as well as other pregnancy complications prompting early delivery (44). The marked differences in GWG between the study groups emphasize the importance of timely and targeted nutritional counseling during pregnancy and highlight the need for greater involvement of healthcare providers in educating women during this vulnerable period. Additionally, these results underscore the necessity of distinguishing between pre-pregnancy body mass index and gestational weight dynamics when assessing prematurity and other pregnancy related risks in women with migraine.

Although initial observations suggested that fetal growth restriction (FGR) was more common in the PTB group, further statistical analysis did not confirm a significant association between prematurity and FGR. FGR was diagnosed in 54 cases, corresponding to an incidence of 14% among fetuses born to mothers with migraine. This exceeds the estimated global FGR incidence of up to 10% (45). While data specifically addressing FGR incidence in women with migraine remain limited, a systematic review and meta-analysis have demonstrated that small for gestational age and low birth weight, both clinical manifestations closely related to FGR, are more frequently observed in pregnancies complicated by migraine (12).

All study participants were affected by migraine, severe chronic neurological condition, and all of them have experienced at least one migraine attack during pregnancy. Despite this shared diagnosis, pregnancy outcomes varied, and migraine characteristics differed significantly between groups. Women in the PTB group experienced more frequent and more intense migraine attacks during pregnancy. This finding represents an important clinical warning signal, highlighting the need for effective migraine symptom control during pregnancy, balancing maternal well-being with fetal safety. A recent meta-analysis reported an association between migraine attacks and adverse pregnancy outcomes, including PTB, and emphasized the need for more effective treatment strategies during pregnancy, while also acknowledging the limited and inconclusive safety data for commonly used medications such as triptans, beta-blockers, and amitriptyline (46).

Importantly, the present findings should be interpreted within the context of the study design. Because all participants were affected by migraine and no non-migraine comparison group was included, the present study cannot establish migraine as an independent causal risk factor for preterm birth. Rather, the results identify vascular and metabolic factors associated with prematurity within a population of pregnant women with migraine.

Several limitations should be acknowledged. First, this was a single-center study conducted at a tertiary referral institution and included only participants from the Serbian population, potentially limiting generalizability. Second, the absence of a non-migraine control group precludes conclusions regarding migraine as an independent risk factor for prematurity. Accordingly, the findings primarily inform risk stratification within a migraine population. Third, migraine subtype stratification (with aura vs. without aura) and detailed treatment data were not consistently available and therefore could not be evaluated. Fourth, although the observed associations support possible vascular and placental mechanisms, the absence of placental histopathology and circulating biomarker analyses limits mechanistic interpretation. Finally, the observational nature of the study does not permit causal inference.

## Conclusion

This study supports the concept that migraine represents a systemic vascular condition associated with adverse pregnancy outcomes. Our findings suggest that pregnant women with migraine may exhibit increased vascular vulnerability, reflected in the strong association between pregnancy-induced hypertension and preterm delivery, as well as increased susceptibility to metabolic and weight-related disturbances. These observations align with established evidence linking migraine to endothelial dysfunction, vascular hyperreactivity, and prothrombotic states, which may compromise maternal cardiovascular adaptation and placental function during pregnancy.

The coexistence of migraine with pregnancy associated hypertension, gestational diabetes mellitus, obesity, and excessive gestational weight gain supports the possibility of an integrated “vascular triad” hypothesis, whereby shared pathophysiological mechanisms, may collectively contribute to increased susceptibility to prematurity and other perinatal complications and poor pregnancy outcomes. While pre-pregnancy obesity alone was not an independent predictor of preterm birth, excessive gestational weight gain emerged as a key modifiable factor, emphasizing the importance of dynamic metabolic changes over static anthropometric measures.

Taken together, our findings underscore the need for individualized, multidisciplinary prenatal care for women affected by migraine, including careful vascular risk assessment, blood pressure monitoring, gestational weight management, and optimized migraine control. Future prospective controlled studies are needed to elucidate the causal pathways linking migraine, placental dysfunction, and adverse pregnancy outcomes, and to establish safe and effective therapeutic strategies that protect both maternal neurological health and fetal well-being.

## Data Availability

The datasets presented in this article are not readily available because data is available upon reasonable request from the first author. Requests to access the datasets should be directed to Milan Lackovic, lackovic011@gmail.com.
